# Biomarkers of Exposure to Polycyclic Aromatic Hydrocarbons and Cognitive Function among Elderly in the United States (National Health and Nutrition Examination Survey: 2001-2002)

**DOI:** 10.1371/journal.pone.0147632

**Published:** 2016-02-05

**Authors:** Elizabeth A. Best, Elizabeth Juarez-Colunga, Katherine James, William G. LeBlanc, Berrin Serdar

**Affiliations:** 1 Department of Epidemiology, Colorado School of Public Health, University of Colorado Denver, Aurora, Colorado, United States of America; 2 Department of Biostatistics and Informatics, Colorado School of Public Health, University of Colorado Denver, Aurora, Colorado, United States of America; 3 Department of Family Medicine, University of Colorado Anschutz Medical Campus, Aurora, Colorado, United States of America; 4 Department of Environmental & Occupational Health, Colorado School of Public Health, University of Colorado Denver, Aurora, Colorado, United States of America; NSYSU, TAIWAN

## Abstract

Recent studies report a link between common environmental exposures, such as particulate matter air pollution and tobacco smoke, and decline in cognitive function. The purpose of this study was to assess the association between exposure to polycyclic aromatic hydrocarbons (PAHs), a selected group of chemicals present in particulate matter and tobacco smoke, and measures of cognitive performance among elderly in the general population. This cross-sectional analysis involved data from 454 individuals aged 60 years and older from the 2001–2002 National Health and Nutrition Examination Survey. The association between PAH exposures (as measured by urinary biomarkers) and cognitive function (digit symbol substitution test (DSST)) was assessed using multiple linear regression analyses. After adjusting for age, socio-economic status and diabetes we observed a negative association between urinary 1-hydroxypyrene, the gold standard of PAH exposure biomarkers, and DSST score. A one percent increase in urinary 1-hydroxypyrene resulted in approximately a 1.8 percent poorer performance on the digit symbol substitution test. Our findings are consistent with previous publications and further suggest that PAHs, at least in part may be responsible for the adverse cognitive effects linked to tobacco smoke and particulate matter air pollution.

## Introduction

Cognitive impairment (CI) is a spectrum condition that primarily affects older populations and involves a decline in thinking abilities including learning new concepts, concentrating, vocalizing, or making decisions [1, 2, 3]. In the United States (U.S.), it is estimated that 20.4 percent of the adult population 65 years and older suffer from some form of CI including Alzheimer’s and dementia [4] which poses social, economic, and medical burden to the society [1]. Mild CI is an early stage of this condition in which cognitive changes are noticeable to the individual and others [5]. The prevalence of mild CI in adults aged 65 years and older is between 10 to 20 percent in the U.S. [6, 7, 8], of which 40 percent is expected to progress into the more severe dementia later in life [9].

DSST, a subtest of the Wechsler Adult Intelligent Test third edition (WAIS-III), is a psychometric assessment used to characterize cognitive function. While not specificin detecting the underlying cause, the DSST is very sensitive in detecting mild CI. A prospective cohort study of elderly French adults demonstrated that the DSST was sensitive to changes in high levels of cognition and was able to detect a one point change in DSST score for those adults who scored 25 or above on the examination [10]. Additionally, Joy et al., 2003, examined 1,167 adults and discovered that visual processing speed and memory, lack of which indicates mild CI, were important predictors in scoring well on the DSST [11]. DSST can reflect short-term effects and has also been widely used to assess acute changes following administration of drugs [12, 13] or to assess the effects of alcohol consumption [14, 15, 16].

Known risk factors for mild CI include increased age and genetic susceptibility [1, 17] while cardiovascular disease (high blood pressure and stroke), diabetes, depression/anxiety, tobacco smoking and social factors (e.g., lack of relationships with others and a community, lack of physical activity, and low socio-economic status (SES)) [1, 17, 18] are reported as possible risk factors. Environmental toxicants, such as polychlorinated biphenyls (PCBs), organochlorine pesticides, and ambient air pollution have also been considered as potential risk factors for CI [19–24]. Recently, research has focused on more specific components of ambient air pollution and particulate matter (PM), such as tobacco smoke and heavy metals [25, 26].

Polycyclic aromatic hydrocarbons (PAHs) are formed through incomplete combustion of organic materials and are widely present in air, soil and water [27, 28, 29]. Exposure to PAHs occurs through inhalation, ingestion and dermal absorption [27]. In the U.S. exposure is more common in urban areas with high traffic and exhaust [27] and in occupations involving coal and gas industries, transportation, and firefighting [27].

There is a small body of evidence that links PAH exposure to neurotoxicity and changes in cognitive function. *In vitro* experiments including animal and human cells report cell death, cell deformities, or decreased cell activity when cells are exposed to PAHs (pyrene or anthracene or benzo(a)pyrene) for a few hours [30–34, 35]. *In vivo* studies report poor performance and recall in rats after short-term exposure to fluorine and naphthalene [36, 37]. Zebrafish exposed to a diet rich in numerous PAHs also performed poorer on various behavioral measures [38].

There is limited indirect evidence that exposure to PAHs can affect cognitive function in humans. PAHs are components of particulate matter (PM), especially in urban areas with higher traffic and automotive exhaust [21, 39]. PM exposure has been linked with CI in humans [21, 22, 23, 24]. While PM between 2.5 and 10 microns in size have been associated with a decrease in cognitive performance [22], PM smaller than 2.5 microns have also been linked to poor verbal learning, executive function and memory among elderly [23]. In a national study of 1,764 adults, every 10 microgram per cubic meter increase in PM levels resulted in a less than 1 unit decrease in DSST scores [24]. More specifically one study reported an association between higher PAH levels in cord blood and increased risk of future attention problems in children [40]. Overall, these consistent findings justify the need to explore the association between PAHs and CI at the population level.

Given the prevalence of PAHs exposures through multiple routes and sources, biomonitoring provides a valuable tool for population based studies. Urinary metabolites have relatively short elimination half-lives ranging between 5–35 hours and are considered useful to estimate recent PAH exposures [41, 42]. Among these, the metabolite of pyrene, 1-hydroxypyrene (1-OHPyr) has been widely accepted as the gold standard biomarker of PAH exposures [43, 44, 45].

This study explores the association between urinary biomarkers of PAHs [1- and 2-hydroxynaphthalene (1-OHN and 2-OHN), 2- and 3-hydroxyfluorene (2-OHFl, 3-OHFl), 1-, 2-, and 3-hydroxyphenanthrene (1-OHPhe, 2-OHPhe, 3-OHPhe), and 1-hydroxypyrene (1-OHPyr)] and mild CI and DSST in a cohort of nationally representative elderly adults from the 2001–2002 National Health and Nutrition Examination Survey (NHANES). Our hypothesis is that there is negative association between urinary PAH metabolite concentrations and DSST score. Given the ubiquitous exposure to PAHs and an aging population prone to an increase in the prevalence of CI, understanding the role of PAHs could be critical for preventing CI or delaying the onset of symptoms.

## Materials and Methods

### Ethics statement

This study used publicly available data, that were anonymized and de-identified prior to analysis, and was exempt from human subjects review.

### Study population and data collection

**Fig 1:**
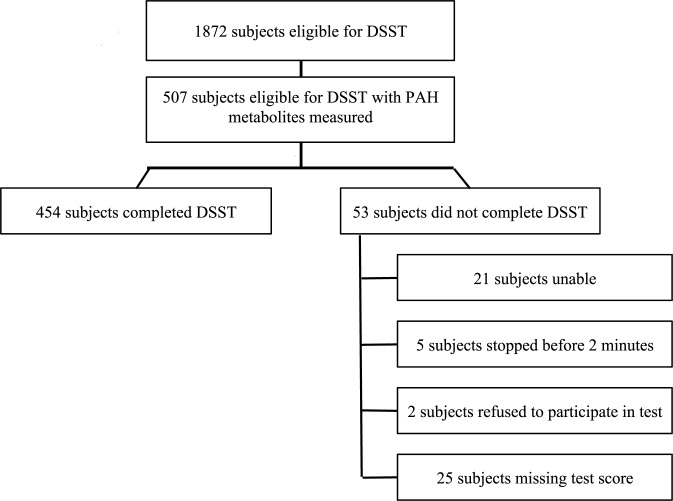
Flow Diagram of the Total Eligible Population and Study Population.

We completed a cross-sectional study using the NHANES 2001–2002. NHANES is an annual repeated cross-sectional survey conducted and maintained by the National Center for Health Statistics of the Center for Disease Control (CDC). NHANES uses a stratified complex sampling scheme to select a nationally representative sample of 5,000 participants per year from 15 randomly selected counties across the US for each cycle to collect clinical, behavioral, demographic, dietary, social, and laboratory data through interviews, exercises, physical examinations, and serum and urine samples. For this study, we used the Continuous NHANES (2001–2002) to select all eligible subjects which included all adults over the age of 60 years with measures for urinary PAH metabolites with a concurrently assessed DSST score (n = 454; Fig 1).

### Cognitive impairment

Cognitive function was assessed by the DSST score, a subtest of the WAIS-III that was administered to all eligible (literate, available writing surface, able to commit to the time frame required to complete the test) adults 60 years and older. This test consists of a coding exercise where individuals print symbols that are matched with numbers, identified in a key, for 133 situations over 120 seconds [46]. The score consists of the number of correct symbols printed during the allotted time frame (minimum: 0 and maximum: 133). DSST score was treated as a continuous variable consistent with previous studies [25, 47].

### Urinary polycyclic aromatic hydrocarbon analysis

The urine samples were collected, stored, and shipped in accordance with the Laboratory/Medical Technologists Procedures Manual. Following collection, the samples were stored at a temperature of -20°C and then sent to National Center for Environmental Health for analysis [48]. Once at the laboratory the urine samples underwent enzymatic hydrolysis to remove PAH conjugates, solid-phase extraction to extract the mono-hydroxylated PAH metabolites, and derivatization to create more volatile metabolites [48]. Urinary metabolites were then quantified using capillary gas chromatography combined with high-resolution mass spectrometry (GC/MS). Method details are presented elsewhere [48]. When urinary PAH metabolite concentrations were non-detectable, a value of the detection limit divided by the square root of two was imputed into the dataset [48].

Levels of urinary PAH metabolites [1-OHN, 2-OHN, 3-OHFl, 2-OHFl, 3-OHPhe, 1-OHPhe, 2-OHPhe, and 1-OHPyr] were evaluated as predictors of the DSST score. Statistical analyses of all PAH metabolite measures were done after natural logarithmic transformation to satisfy normality assumption (S1 Appendix). Urinary PAHs were assessed in three separate measures: 1) sum of all urinary PAH metabolites; 2) sum of smaller molecular PAHs (NFP) consisting of naphthols (1-OHN, 2-OHN), fluorenols (2-OHFl, 3-OHFl), and phenanthrols; and 3) measures of 1-pyrenol (1-OHPyr). Each of these biomarker measures was evaluated as a predictor of DSST score in separate linear regression models.

### Other risk factors

Potential confounders to the association between urinary PAH metabolites and DSST scores were evaluated based on findings from previous research. These included age, gender, SES [18], alcohol consumption [14], smoking status [25], vision and hearing problems [49], hypertension diagnosis [50], physical activity [51], history of thyroid disease [52], history of kidney disease [53], history of liver disease, history of stroke [54], current use of medications that potentially alter test taking ability such as medication to treat depression [55], anxiety [56], dementia/Alzheimer’s, and pain killers [57]. In this study, test taking impairment was defined as having poor self-reported hearing, vision, or both poor vision and hearing. Medication that could potentially alter test taking performance was investigated by combining the use (user/non-user) of the following FDA drug classes: anti-depressant, anti-anxiety, dementia, Alzheimer’s disease, and pain killers. Clinical data was collected through an examination and medical interview. Subjects were asked to self-report previous clinical diagnoses (yes/no) including diseases of the kidney, liver, thyroid, and stroke. Blood pressure was measured four times for each individual in a seated position, after 5 minutes of rest using a sphygmomanometer. Hypertension (yes/ no) was assigned to each individual if his/her average systolic blood pressure was higher than140 mmHg, average diastolic blood pressure was higher than 90 mmHg, or both. Urinary creatinine was measured via gas GC/MS for each subject. Biomarkers in spot urine samples are variable due to changes in urine dilution. To adjust for this variation, urinary creatinine measurements were evaluated as potential confounders in linear regression models [58].

Study subjects were interviewed regarding demographic factors (e.g., age, gender, SES), lifestyle, and medical conditions. Demographic information including age, gender, and SES were collected. Age was recorded for each study subject as a continuous variable up to age 84. All individuals who were 85 and older were categorized into one age category defined as 85 and older. SES was estimated based on the family income poverty index ratio (ratio of the family income to poverty threshold as reported by the Department and Health and Human Services) and categorized into three groups low (0–1.85), middle (1.85–3.5), and high (3.5-above).

Behavioral information was collected through a medical interview. Subjects were asked about current alcohol consumption, and were defined as drinkers (more than 12 alcoholic drinks a year) or non-drinkers (fewer than 12 alcoholic drinks per year) to assess the interaction between alcohol and urinary metabolite levels, as found in previous studies [59, 60]. Tobacco smoke exposure was defined as having smoked 100 cigarettes or more over one’s lifetime, smoked 20 cigars over one’s lifetime, or smoked 20 pipes over one’s lifetime (yes/no). Physical activity level (low, medium, high) was determined through a physical assessment algorithm derived from interview questions about duration, intensity, and type of exercise.

Non-valid measures of variables included missing values or responses of “I don’t know” or “refuse to answer”. Non-valid measures were reported for SES, alcohol consumption, physical activity, thyroid issues, stroke, kidney disease and liver disease.

### Statistical analysis

All analyses were conducted using SAS Version 9.4 using survey procedures (PROC SURVEY REG) that account for the complex survey design. Missing data labeled as ‘non-valid response’ for alcohol consumption, physical activity level, thyroid disease, and stroke were imputed by assigning the value of the highest frequency from available valid responses. A two-step algorithm was used to impute the missing SES values. SES was imputed as low for individuals with individual family income less than $20,000 per year, Medicaid use, or marginally secure food security. The remaining SES values were imputed with the highest SES frequency by education status for each of the missing individuals. Residuals and outliers were evaluated for the outcome and primary exposure variable with residual plots (S2 Appendix) and a criterion of less than 5 percent presence was determined to be acceptable. Two values for 1-OHN and 2-OHN were high in our final study population. To evaluate their influence these observations were removed from the model and analyses were repeated. Results for both models were similar (with two high values in data: parameter estimate of 1-OHPyr = -1.81 (95 percent confidence interval of -3.41, -0.21), and model r-squared of 0.366; and without the two high values: parameter estimate of 1-OHPyr = -1.82 (95 percent confidence interval of -3.44, -0.20), and model r-squared of 0.365).

A non-response analysis was performed to confirm that those who were eligible for the DSST but did not have a valid measure were similar to those who did complete the DSST. To test if there were significant differences in the three urinary PAH metabolite measures amongst covariate categories, means were compared with a t-test using PROC SURVEY REG with a LSMEANS statement. Additionally, the Pearson correlation was calculated for each of the three urinary PAH metabolite measures and the DSST score in univariate analyses (using PROC CORR).

Univariate linear regression (using PROC SURVEY REG) investigating the association of urinary PAH metabolite concentrations and other covariates and DSST score were conducted to assess potential variables in a multiple regression model. Significance for the univariate analyses was defined as a p-value less than 0.1. Collinearity was assessed for all covariates identified during the univariate analyses.

Multiple linear regression modeling (using PROC SURVEY REG) was used to assess the association between urinary PAH metabolite concentrations and DSST score. The general multiple linear regression model is: y = β_o_ + β_1_log(X_1_) +β_2_log(X_2_) … + e; for our study y represents the subject specific DSST score, X_1_ represents the subject specific PAH concentrations, β_1_ is a regression coefficient which represents the change in the mean DSST score corresponding to a unit change in the log transformed subject specific PAH concentration(s), X_2_ denotes other covariates to adjust for in the model, with corresponding regression coefficient β_2,_ and e is the error term. A backwards method was used to determine the factors of DSST score. During the backward selection process, all significant variables determined in the univariate analyses were entered into the model and the variable with the highest p-value was removed with each iteration until the p-values for all remaining variables were less than 0.05. Factors with a p-value of less than 0.05 were retained in final models. A sensitivity analysis was conducted to determine if similar results were obtained with and without imputation of non-valid or missing covariates.

## Results

### Descriptive statistics

Residuals plots were analyzed and no clear violations of the linear regression assumptions were observed. Table 1 presents geometric mean and geometric standard error for the final study population, the non-response population (those who were eligible to complete the DSST but who did not have a score), and for the eligible study population. As can be seen in Table 1, the eligible study population without a DSST score (n = 53) had a higher percentage of participants who were classified as low SES, report drinking alcohol, report tobacco use, and suffer from hearing and vision impairment. The non-response population had higher concentrations of the sum of all urinary PAH metabolites and NFP compared to the final study population as was indicated by mean difference t-tests with p-values of <0.001 for both.

**Table 1:** Population Statistics for Study Cohort.

	Study Population (n = 454)	Non-Response Population (n = 53)	Total Eligible Population (n = 507)
	**n (%) or Mean (SE)**	**n (%) or Mean (SE)**	**n (%) or Mean (SE)**
**Demographic Characteristics**			
**Age**	70.1 (0.520)	73.2 (1.03)	70.4 (0.500)
**Gender**			
Male	221 (48.7)	24 (45.3)	245 (48.3)
Female	233 (51.3)	29 (54.7)	262 (51.7)
**Socio-Economic Status**^1^			
Low	165 (36.3)	27 (50.9)	192 (37.9)
Middle	116 (25.6)	12 (22.6)	128 (25.2)
High	143 (31.5)	6 (11.3)	149 (29.4)
Missing	30 (6.61)	8 (15.1)	38 (7.50)
**Lifestyle Characteristics**			
**Alcohol Use**			
Yes	266 (58.6)	28 (52.8)	294 (58.0)
No	170 (37.4)	16 (30.2)	186 (36.7)
Missing	18 (3.96)	9 (17.0)	27 (5.30)
**Smoking Status**			
Smoker	255 (56.2)	33 (62.3)	288 (56.8)
Non-smoker	199 (43.8)	20 (37.7)	219 (43.2)
**Physical Activity**			
Low	135 (29.7)	29 (54.7)	164 (32.3)
Moderate	246 (54.2)	20 (37.7)	266 (52.5)
High	71 (15.6)	4 (7.6)	75 (14.8)
Refused to answer	1 (0.220)	-	1 (0.200)
I don't know	1 (0.220)	-	1 (0.200)
**Test Taking Conditions**^2^			
Impaired	382 (84.1)	50 (94.3)	432 (85.2)
Non-impaired	72 (15.9)	3 (5.66)	75 (14.8)
**Clinical Conditions**			
**Blood Pressure**^3^			
Hypertensive	184 (40.5)	24 (45.3)	208 (41.0)
Non-hypertensive	270 (59.5)	29 (54.7)	299 (59.0)
**Thyroid Issues**			
Yes	66 (14.5)	3 (5.66)	69 (13.6)
No	387 (85.2)	50 (94.3)	437 (86.2)
I don't know	1 (0.220)	-	1 (0.20)
**Stroke**			
Yes	27 (5.95)	6 (11.3)	33 (6.51)
No	426 (93.8)	47 (88.7)	473 (93.3)
I don't know	1 (0.220)	-	1 (0.200)
**Kidney Disease**			
Yes	15 (3.30)	4 (7.55)	19 (3.75)
No	437 (96.3)	49 (92.5)	486 (95.9)
I don't know	2 (0.440)	-	2 (0.390)
**Liver Disease**			
Yes	13 (2.86)	-	13 (2.56)
No	439 (96.7)	52 (98.1)	491 (96.8)
I don't know	2 (0.440)	1 (1.89)	3 (0.590)
**Diabetes**			
Yes	75 (16.5)	10 (18.9)	85 (16.8)
No	364 (80.2)	42 (79.3)	406 (80.1)
Borderline	15 (3.30)	1 (1.89)	16 (3.16)
**Medication Use Potentially Altering Cognitive Ability**^4^			
Use	16 (3.52)	3 (5.66)	19 (3.75)
Non-use	438 (96.5)	50 (94.3)	488 (96.3)
** **	**Mean (SE)**	**Mean (SE)**	**Mean (SE)**
**Digit Symbol Substitution Test Score**	44.1 (18.5)	-	44.1 (18.5)
0–26	80 (17.6)	-	80 (17.6)
27–61	302 (66.5)	-	302 (66.5)
62+	72 (15.9)	-	72 (15.9)
** **	**GM (GSE)**	**GM(GSE)**	**GM (GSE)**
**Total PAHs**^5^ **(ng/L)**	5830 (1.08)	8350 (1.90)	6250 (1.32)
**Naphthols + Fluorenols + Phenanthrols**^5^ **(ng/L)**	5650 (1.08)	7040 (1.27)	5770 (1.07)
**1-OHPyr**^5^ **(ng/L)**	32.1 (1.06)	34.8 (1.16)	32.5 (1.05)
**Creatinine**^5^ **(mg/dL)**	83.1 (1.03)	92.8 (1.88)	86. 5 (2.01)

^1^The socio-economic variable was developed by categorizing the family poverty index ratio into three categories: 0–1.85 (low), 1.851–3.500 (middle), and 3.501-above (high).

^2^The test taking conditions variables were developed by categorizing those who had a vision problem, hearing problem, or both as impaired and those who did not have a vision problem, hearing problem, or both as non-impaired. The questionnaire questions used to ascertain the data to make these categories were: "Is your hearing described as good, little trouble, lot of trouble or deaf?" and "Do you usually require glasses to read?".

^3^Hypertension was determined by an average systolic blood pressure of >140 mmHg, an average diastolic blood pressure of >90 mmHg, or both. Four blood pressure measurements were obtained and averaged for each individual during the examination.

^4^These medications include those classified with a FDA class code of antidepressive, anti-anxiety, dementia, Alzheimer's Disease, or pain reliever.

^5^Geometric means are presented.

n- number

%—percent

GM–geometric mean

GSE–geometric standard error

SE—standard error

PAHs—polycyclic aromatic hydrocarbons

ng/L—nanograms per liter

mg/dL—milligrams per deciliter

1-OHPyr—1-hydroxypyrene

Tables 2 and 3 present descriptive statistics for the main explanatory variables and covariates in the final study population. PAH metabolites were quantified in most of the population, with less than 5 percent non-detect for each PAH metabolite. The geometric means for the total PAH, NFP, and 1-OHPyr urinary metabolite concentrations were 5,830 nanograms per liter (ng/L), 5,650 ng/L, and 32.1 ng/L, respectively (Table 2). Participants with middle and upper SES had the lowest PAH metabolite concentrations, while subjects of lower SES had the highest PAH concentrations (Table 3). Additionally, subjects who smoked had higher urinary PAH metabolite concentrations compared to non-smokers (Table 3). The correlation between the urinary PAH metabolite concentrations and the DSST scores were low and in the negative direction, with Pearson correlation coefficients around -0.08 for all and p-values between 0.072–0.107 (Table 2). The highest DSST scores were observed for those classified as high SES; the lowest DSST scores were observed for those of low SES.

**Table 2:** Means and Standard Errors for Continuous Variables.

Continuous Variables							
	Mean (SE)	min	Q1	Median	Q3	max	Pearson r^1^
**Total PAHs**^2^ **(ng/L)**	5830 (1.08)	219	2370	5380	11500	299000	-0.080 (0.08)
**Naphthols + Fluorenols + Phenanthrols**^2^ **(ng/L)**	5650 (1.08)	217	1960	5220	11300	299000	-0.080 (0.08)
**1-OHPyr**^2^ **(ng/L)**	32.1 (1.06)	2.10	16.6	33.1	59.5	1860	-0.080 (0.11)
**Creatinine**^2^ **(mg/dL)**	83.1 (1.03)	5.99	53.0	95.6	138	384	-0.004 (0.92)
**Age**	70.1 (0.52)	60.0	63.5	63.4	74.9	85.0	-0.320 (<0.0001)

^1^Correlation of total PAHs, age, and creatinine with cognitive function score and corresponding p-value in parentheses.

^2^Geometric means are presented.

SE—standard error

min—minimum

Q1—1st quartile

Q3—3rd quartile

max—maximum

PAHs—polycyclic aromatic hydrocarbons

ng/L—nanograms per liter

mg/dL—milligrams per deciliter

1-OHPyr—1-hydroxypyrene

**Table 3:** Means and Standard Errors for Categorical Variables by DSST and PAH Metabolite Groupings.

Categorical Variables				
	Digit Symbol Substitution Test Score	Total PAHs^1^ (ng/L)	N,F,P^1^ (ng/L)	1-OHPyr^1^ (ng/L)
	Mean (SE)	GM (GSE)	GM (GSE)	GM (GSE)
**Gender**				
Male	47.4 (1.06)	6840 (1.12)	6770 (1.12)	41.7 (1.09)
Female	49.2 (1.36)	5010 (1.08)	5010 (1.08)	26.6 (1.08)
**SES**^2^				
Low	36.1 (1.51)	7630 (1.12)	7550 (1.12)	39.3 (1.12)
Middle	49.4 (1.56)	4320 (1.12)	4270 (1.12)	28.5 (1.09)
High	58.1 (1.37)	5600 (1.09)	5540 (1.09)	29.7 (1.11)
**Alcohol Use**				
Yes	49.5 (1.23)	6440 (1.08)	6440 (1.08)	35.9 (1.07)
No	46.7 (1.48)	4720 (1.09)	4670 (1.12)	26.8 (1.08)
**Smoking Status**				
Smoker	48.5 (1.09)	7480 (1.13)	7400 (1.08)	41.3 (1.06)
Non-smoker	48.3 (1.37)	4150 (1.08)	4100 (1.13)	23.6 (1.09)
**Physical Activity**				
Low	41.5 (2.65)	7410 (1.17)	7330 (1.17)	33.8 (1.13)
Moderate	49.6 (1.17)	5120 (1.08)	5060 (1.08)	30.0 (1.08)
High	55.8 (2.35)	5430 (1.22)	5380 (1.22)	36.6 (1.16)
**Test Taking Conditions**^3^				
Impaired	47.9 (1.03)	6000 (1.08)	5940 (1.08)	34.8 (1.07)
Non-impaired	50.9 (2.77)	4820 (1.23)	4770 (1.23)	22.9 (1.22)
**Blood Pressure**^4^				
Hypertensive	45.6 (1.41)	6310 (1.08)	6250 (1.08)	34.5 (1.08)
Non-hypertensive	50.1 (1.03)	4910 (1.08)	4870 (1.08)	28.8 (1.08)
**Thyroid Issues**				
Yes	49.7 (2.65)	4910 (1.16)	4870 (1.16)	26.8 (1.11)
No	48.2 (0.68)	5940 (1.09)	5880 (1.09)	33.5 (1.07)
**Stroke**				
Yes	36.8 (2.79)	3900 (1.27)	3870 (1.27)	30.6 (1.08)
No	49.9 (0.90)	5880 (1.08)	5830 (1.08)	32.5 (1.07)
**Kidney Disease**				
Yes	35.8 (5.38)	8100 (1.30)	8020 (1.30)	48.9 (1.22)
No	48.9 (0.90)	5710 (1.08)	5650 (1.08)	32.1 (1.09)
**Liver Disease**				
Yes	46.7 (5.49)	7710 (1.45)	7630 (1.45)	32.8 (1.06)
No	48.4 (0.91)	5650 (1.08)	5650 (1.08)	18.5 (1.75)
**Diabetes**				
Yes	40.7 (2.15)	5060 (1.14)	5010 (1.14)	26.6 (1.14)
No	49.7 (1.07)	5940 (1.09)	5880 (1.09)	33.5 (1.07)
Borderline	54.1 (4.94)	4630 (1.39)	4580 (1.39)	28.5 (1.57)
**Medications Use Potentially Altering Cognitive Ability**^5^				
Use	45.9 (4.30)	9050 (1.19)	8960 (1.19)	33.1 (1.14)
Non-use	48.5 (0.92)	5650 (1.08)	5650 (1.08)	32.1 (1.06)

^1^Geometric means are presented.

^2^The socio-economic variable was developed by categorizing the family poverty index ratio into three categories: 0–1.85 (low), 1.851–3.500 (middle), and 3.501-above (high).

^3^The test taking conditions variables were developed by categorizing those who had a vision problem, hearing problem, or both as impaired and those who did not have a vision problem, hearing problem, or both as non-impaired. The questionnaire questions used to ascertain the data to make these categories were: "Is your hearing described as good, little trouble, lot of trouble or deaf?" and "Do you usually require glasses to read?".

^4^Hypertension was determined by an average systolic blood pressure of >140 mmHg, an average diastolic blood pressure of >90 mmHg, or both. Four blood pressure measurements were obtained and averaged for each individual during the examination.

^5^These medications include those classified with a FDA class code of antidepressive, anti-anxiety, dementia, Alzheimer's Disease, or pain reliever.

GM–geometric mean

GSE–geometric standard error

SE—standard error

PAHs—polycyclic aromatic hydrocarbons

N,F,P—naphthols, fluorenols, and phenanthrols

ng/L—nanograms per liter

1-OHPyr—1-hydroxypyrene

Vision and hearing impairment were not considered for further univariate or model selection analyses as the majority (84 percent) of the study population was considered impaired. Kidney disease, liver disease, and medication use were also not considered for the univariate or model selection analyses as the majority of the study population (greater than 95 percent) were classified as not having kidney disease, liver disease, or not taking cognitive altering medications.

### Univariate analyses

Table 4 presents the univariate associations between the DSST score and each covariate of interest. All PAH metabolite explanatory variables were significantly associated with the DSST score. Additionally, age (continuous), SES, physical activity, blood pressure, stroke, and diabetic status were also significantly associated with DSST score. Older age, lower SES, limited physical activity, previous stroke, and physician diagnosed diabetes were associated with poorer performance on the DSST. Conversely, current hypertension was associated with better performance on the DSST.

**Table 4:** Parameter Estimates (95% CIs) and Coefficients of Determination of Digit Symbol Substitution Test Scores by All Covariates from Univariate Analyses.

	Parameter Estimate	p-Value	r^2^
**Age**	-1.00 (-1.24, -0.760)	<0.0001*	0.159
**Gender**		0.326	0.002
Male	-1.83 (-5.67, 2.01)		
Female	-		
**SES**		<0.0001*	0.257
Low	-22.0 (-27.0, -17.0)		
Middle	-8.74 (-13.2, -4.32)		
High	-		
**Alcohol Use**		0.195	0.005
Yes	2.78 (-1.59, 7.14)		
No	-		
**Smoking Status**		0.873	0.00005
Smoker	-0.270 (-3.82, 3.28)		
Non-smoker	-		
**Physical Activity**		0.009*	0.073
Low	-14.3 (-22.9, -5.64)		
Moderate	-6.17 (-11.1, -1.22)		
High	-		
**Blood Pressure**		0.013*	0.014
Hypertensive	4.52 (1.11, 7.92)		
Non-hypertensive	-		
**Thyroid Issues**		0.531	0.0009
Yes	1.51 (-3.52, 6.54)		
No	-		
**Stroke**		0.0003*	0.023
Yes	-12.3 (-18.0, -6.60)		
No	-		
**Diabetes**		0.013*	0.031
Yes	-13.4 (-27.4, 0.650)		
No	-4.53 (-15.4, 6.34)-		
Borderline			
**Total PAHs**^**1**^	-2.03 (-4.22, 0.170)	0.068*	0.018
**Naphthols + Fluorenols + Phenanthrols**^**1**^	-2.02 (-4.21, 0.180)	0.069*	0.018
**1-OHPyr**^**1**^	-1.93 (-3.82, -0.040)	0.046*	0.013
**Creatinine**^**1**^	-1.25 (-4.29, 1.80)	0.396	0.002

^1^Log transformed.

* significant at the 0.10 level

1-OHPyr—1-hydroxypyrene

### Multiple linear regression

Linear regression models for each PAH explanatory variable are presented in Table 5. After adjusting for age, SES, and diabetic status, we observed a negative association between DSST score and total urinary PAH metabolites, as well as with NFP urinary metabolites, which were not statistically significant (p-value 0.053 for both models). For every 1 percent increase in 1-OHPyr in urine, however, we observed a statistically significant decrease of 1.81% in DSST score after adjusting for age, SES, and diabetic status (p-value 0.029).

**Table 5:** Parameter Estimates (95% CIs) and Coefficient of Determination of Digit Symbol Substitution Test Scores by Final Model Covariates^1^ for each PAH Metabolite Grouping.

**Total PAHs**			
** **	**Parameter Estimates**	**p-Value**	**Final Model r**^**2**^
**PAH Metabolite(s)**^2^	-1.70 (-3.41, 0.020)	0.053	0.367
**Age**	-0.730 (-0.93, -0.530)	<0.0001*	
**SES**		<0.0001*	
Low	-17.5 (-21.9, -13.2)		
Middle	-6.90 (-11.4, -2.45)		
High	-		
**Diabetes**		0.004*	
Yes	-13.4 (-25.7, -1.04)		
No	-4.50 (-14.8, 5.76)		
Borderline	-		
Intercept	128.0 (107, 149)	<0.0001*	
**Naphthols, Fluorenols, & Phenanthrols**			
** **	**Parameter Estimates**	**p-Value**	**Final Model r**^**2**^
**PAH Metabolite(s)**^2^	-1.69 (-3.40, 0.030)	0.053	0.367
**Age**	-0.730 (-0.930, -0.530)	<0.0001*	
**SES**		<0.0001*	
Low	-17.5 (-21.9, -13.2)		
Middle	-6.90 (-11.4, -2.45)		
High	-		
**Diabetes**		0.004*	
Yes	-13.4 (-25.7, -1.04)		
No	-4.54 (-14.8, 5.76)		
Borderline	-		
Intercept	128 (107, 148)	<0.0001*	
**1-OHPyr**			
** **	**Parameter Estimates**	**p-Value**	**Final Model r**^**2**^
**PAH Metabolite(s)**^2^	-1.81 (-3.41, -0.210)	0.029*	0.366
**Age**	-0.740 (-0.940, -0.540)	<0.0001*	
**SES**		<0.0001*	
Low	-17.5 (-21.9, -13.1)		
Middle	-6.50 (-10.6, -2.40)		
High	-		
**Diabetes**		0.004*	
Yes	-13.6 (-26.0, -1.19)		
No	-4.56 (-15.3, 6.14)		
Borderline	-		
Intercept	120 (104, 136)	<0.0001*	

^1^Age, SES, physical activity, blood pressure, stroke, and diabetic status were significantly associated with DSST scores during the univariate analyses and were considered during multiple linear regression using a backward selection process;-all remaining variables were <0.05.

^2^Log transformed.

* significant at the 0.05 level

1-OHPyr—1-hydroxypyrene

Urinary biomarkers are usually adjusted for urine dilution through urinary creatinine by either directly dividing biomarker measurements with creatinine levels or keeping urine creatinine as a predictor in final models.[53] In our analyses urinary creatinine was not a significant predictor in final models and its addition did not alter the root mean squared error in any of the models of total PAHs, NFPs, and 1-OHPyr, respectively. Thus, urinary creatinine was not kept in final models (data not shown.).

### Sensitivity analysis

To examine the effect of imputation, the final models were re-analyzed with non-valid response and missing values omitted from the dataset (n = 403). The root mean squared errors decreased by less than 2 percent between the imputed and non-imputed final models for all three groups of PAH metabolites. As the decrease in root mean squared errors was negligible, we chose to use the imputed dataset to increase the power. With a sample size of 400 to 450 and an alpha level of 0.05, the detectable percentage of the variance explained by a predictor on the DSST score using multiple regression with 4 predictors is 0.018–0.020 with a power of 0.8 (data of sensitivity analysis not shown).

## Discussion

This study examines the association between urinary PAH metabolites and DSST scores among a nationally representative sample of elderly Americans. We observed a negative association between urinary 1-OHPyr levels and measures of cognitive function. A similar association was observed for the models of sum of PAH metabolites and the combination of naphthols, fluorenols, and phenanthrols, but these did not reach statistical significance. SES yielded the largest parameter estimates in the final models examining predictors of DSST score. This is consistent with a previous study that showed SES (measured by the poverty index ratio) was independently associated with cognitive function (DSST) for those older than 60 in two survey cycles of NHANES [18]. SES was also considered to be an important predictor in studies that evaluated other environmental contaminants (such as pesticides and PCBs) as predictors of DSST, but we are unable to compare our results since parameter estimates were not provided [19, 20].

One recent study assessed the association between blood cotinine levels, a marker of nicotine exposure, and the DSST score in elderly non-smokers using data from the 1999–2002 NHANES [25]. While blood cotinine measurements were not available in our study population, the regression coefficients for PAH metabolites observed in our final models are similar to those observed for blood cotinine levels in the Akhtar study. In our study, DSST scores were lower by 1.69–1.81 points per one unit increase in log-transformed PAH metabolite levels. Akhtar et al. reported that DSST scores were lower by 1.17–2.03 and 0.91–0.93 points per one unit increase in log-transformed PAH metabolite levels, for never and former smokers, respectively [25]. Despite this consistency there were some differences between the two studies since Akhtar et al. excluded current smokers from their analysis and adjusted for many additional variables that were not significant in our models (gender, race/ethnicity, hypertension, body mass index, alcohol, stroke and heart attack). Yet, the consistency of our results is noteworthy. The association between cotinine, a metabolite of nicotine, and DSST score may in fact reflect the effects of specific chemicals in tobacco smoke, such as PAHs. Furthermore, previous studies have suggested a positive association between nicotine and cognitive performance. A meta-analysis of more than 20 studies reported mostly positive associations between nicotine and cognitive function [61]. However, only two of these studies were in human subjects and involved administration of nicotine via an injection rather than nicotine exposure via tobacco use [62, 63]. Therefore, more research is needed to parse out the observed effects of tobacco smoke on cognitive function.

Our study has several limitations that should be considered when interpreting our results. The causal nature of our study design should be interpreted with caution as the cross-sectional study lacks temporality. PAH metabolites are eliminated from the body within one day [41, 42] and therefore, PAH metabolite biomarkers represent recent exposures rather than long term exposures. DSST is a highly sensitive test that has been widely used to evaluate not just chronic changes in cognitive function, but also acute alterations due to short-term chemical exposures, such as alcohol consumption [14, 15, 16]. There is some evidence from animal studies that short-term PAH exposures lead to acute neurological effects, such as decreased performance on functional observation battery exam or behavioral changes in rats [33, 36]. Thus, the negative association observed between PAH metabolites and DSST measures in this study may very well represent acute effects of short-term PAH exposures. As PAH exposures are likely to be consistent over longer periods in this elderly group of the general population, it is reasonable to assume that our results may also reflect effects of longer-term exposures.

As the non-response population had higher concentrations of the sum of all urinary PAH metabolites (mean difference t-test p-value of <0.001) and NFP (mean difference t-test p-value of <0.001) compared to the final study population, a non-response bias may be present and the association between higher PAH concentrations and poorer DSST scores in our final model may be an underestimate with the omission of these 53 individuals. However, there was not a significant difference in mean urinary PAH metabolite concentrations between smokers in the final study population compared to smokers in the non-response population (mean difference t-test p-values of 0.456 for the sum of all urinary metabolites, 0.457 for NFP, and 0.790 for 1-OHPyr). Absence of a significant difference in urinary PAH metabolite concentrations between non-smokers in the final study population compared to non-smokers in the non-response population minimizes the likelihood of a non-response bias.

The main strengths of this study are the use of the highly generalizable NHANES data, the fulfillment of a data gap, and the use of biomarkers of exposure to a specific chemical group. The NHANES is designed to be a nationally representative, thus the results from our study are potentially generalizable across the U.S. population. However, this should be done with caution as our sample size is a small fraction of the NHANES cycle. The use of biomarkers in our study provides an internal dose for exposure to PM air pollution rather than aggregated measurements from environmental monitoring. To the best of our knowledge, this is the first study to investigate the association between PAH exposure and cognitive function in an elderly human population of this magnitude. 1-OHPyr has been identified as a reliable and useful biomarker of PAH exposures, particularly exposures to PAHs related to PM and air pollution [25, 43, 44, 64]. While studies have linked PM air pollution to impaired cognitive function, our results present an association with a specific component of PM air pollution.

## Conclusions

Urinary 1-OHPyr, the gold standard of PAH exposure, was significantly associated with poorer cognitive performance in a representative sample of the U.S. elderly population. Our findings are consistent with a previous study that tobacco smoke exposure may be responsible for lower cognitive performance. It is possible that PAHs are, at least in part, responsible for adverse cognitive effects of tobacco smoke. PAHs are recognized for their health risks, including carcinogenic effects, yet their impact on cognitive performance is not well known. Our results support that further studies are needed to understand adverse effects of this widely distributed environmental pollutant on cognitive performance in vulnerable groups, such as the elderly population.

## Supporting Information

S1 Appendix Scatterplots of polycyclic aromatic hydrocarbons (PAHs) and the digit symbol substitution test (DSST) scores before and after logarithmic transformation of urinary biomarkers. (DOCX)

S2 Appendix Residual plots for the outcome and primary exposure variables. (DOCX)

## Abbreviations

CI: cognitive impairment
U.S.: United States
SES: socio-economic status
PCBs: polychlorinated biphenyls
PM: particulate matter
PAHs: polycyclic aromatic hydrocarbons
DSST: digit symbol substitution test
WAIS-III: Wechsler Adult Intelligent Test
1-OHN: 1-hydroxynaphthalene
2-OHN: 2-hydroxynaphthalene
2-OHFl: 2-hydroxyfluorene
3-OHFl: 3-hydroxyfluorene
1-OHPhe: 1-hydroxyphenanthrene
2-OHPhe: 2-hydroxyphenanthrene
3-OHPhe: 3-hydroxyphenanthrene
1-OHPyr: 1-hydroxypyrene
NHANES: National Health and Nutrition Examination Survey
CDC: Center for Disease Control
GC/MS: gas chromatography combined with high-resolution mass spectrometry
ng/L: nanograms per liter
