# Supplementary material for: Biomarkers of Exposure to Polycyclic Aromatic Hydrocarbons and Cognitive Function among Elderly in the United States (National Health and Nutrition Examination Survey: 2001-2002)
Source: PLoS One. 2016 Feb 5;11(2):e0147632. doi: 10.1371/journal.pone.0147632 (PMC4744008; doi:10.1371/journal.pone.0147632)
Supplement: S1 Appendix — (DOCX) [file pone.0147632.s001.docx]

**S1 Appendix**

**Plot 1**: Concentration of total polycyclic aromatic hydrocarbons (PAHs) on the left and concentration of total PAHs, log transformed, on the right compared to digit symbol substitution test (DSST) scores


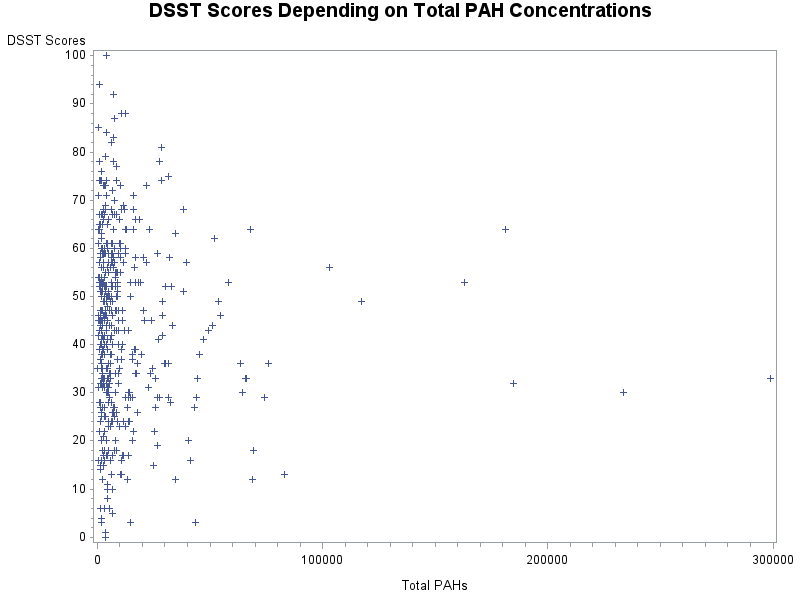


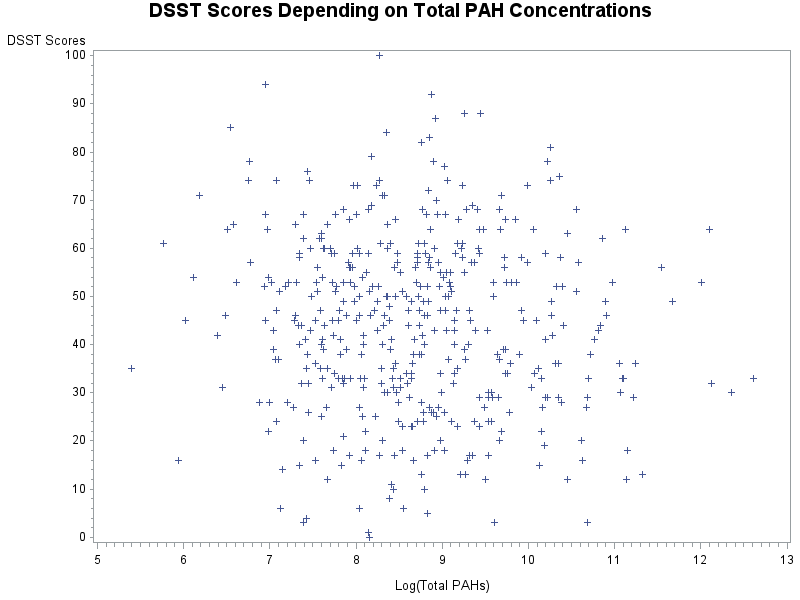


**
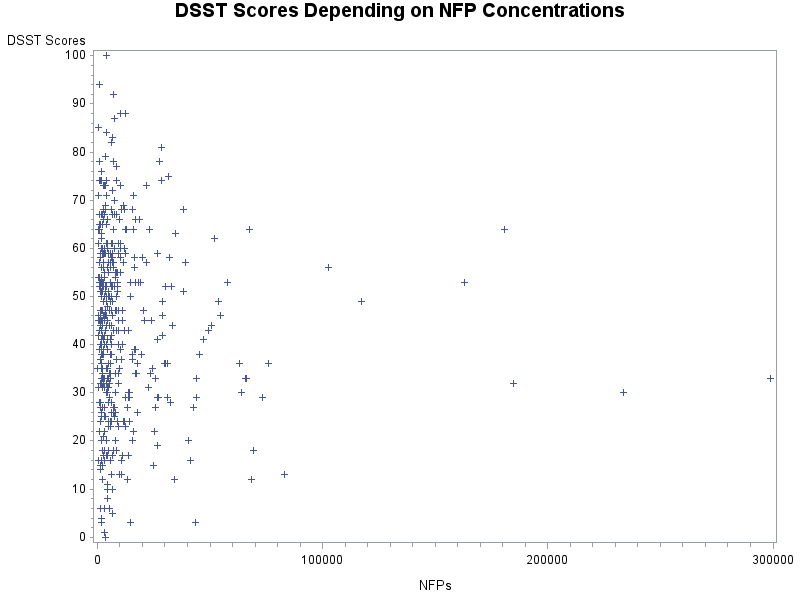
Plot 2**: Concentration of the summation of naphthols, fluorenols, and phenanthrols (NFPs) on the left and concentration of NFPs, log transformed, on the right compared to the DSST scores

**
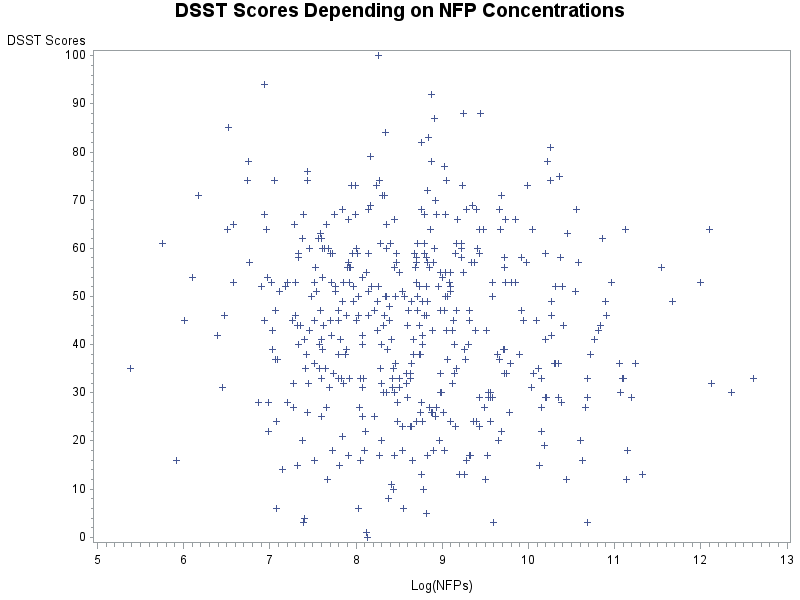
**

**
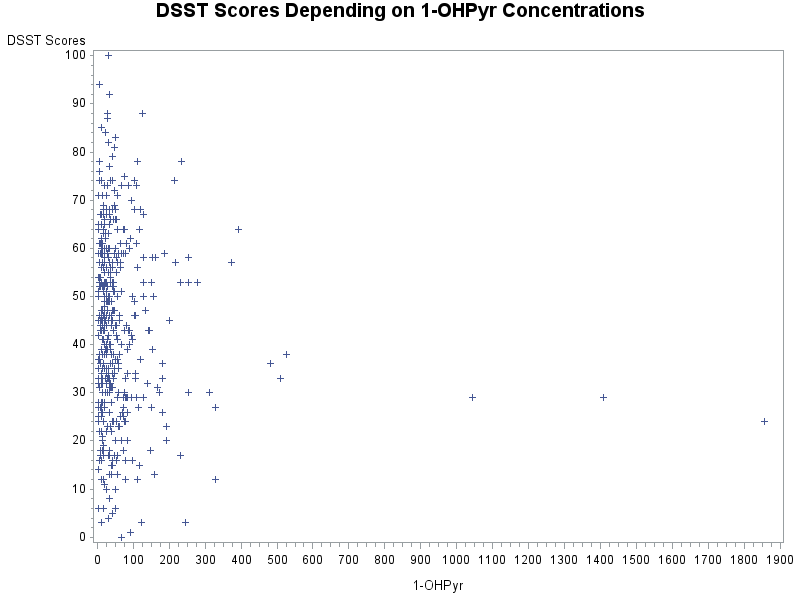
Plot 3**: Concentration of 1-hydroxypyrene (1-OHPyr) on the left and concentration of 1-OHPyr, log transformed, on the right compared to the DSST scores

**
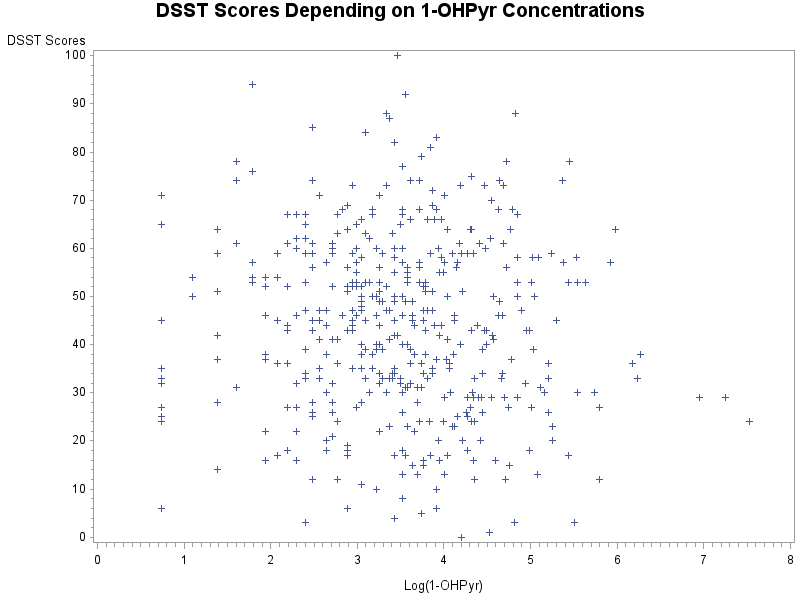
**
