# Supplementary material for: Biomarkers of Exposure to Polycyclic Aromatic Hydrocarbons and Cognitive Function among Elderly in the United States (National Health and Nutrition Examination Survey: 2001-2002)
Source: PLoS One. 2016 Feb 5;11(2):e0147632. doi: 10.1371/journal.pone.0147632 (PMC4744008; doi:10.1371/journal.pone.0147632)

**S2 Appendix**

**Model 1: Digit symbol substitution test (DSST) scores compared to total polycyclic aromatic hydrocarbon (PAH) concentrations, log transformed, and adjusted for age, socio-economic status, and diabetic status**

**Plot 1:** Distribution and probability plot, in normal quantiles, for model residuals


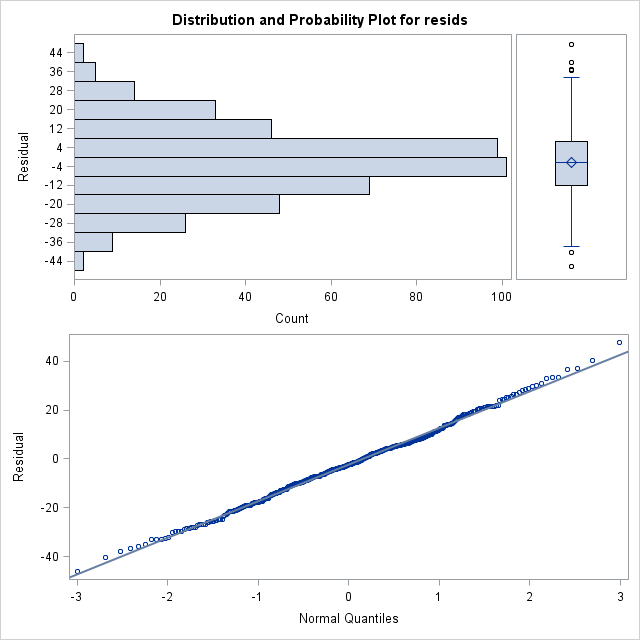


**Plot 2:** Model residuals compared to model predicted values


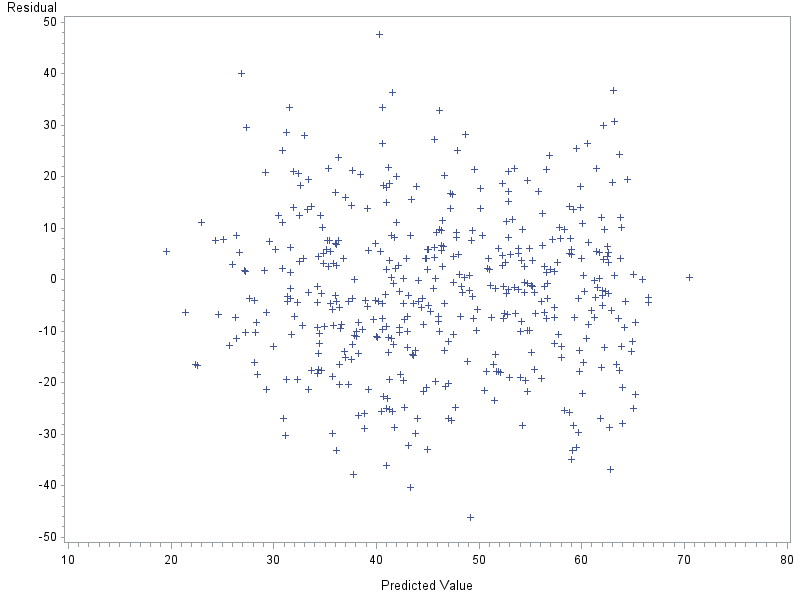


**Plot 3:** Predicted and observed DSST scores compared to log transformed total PAH concentrations


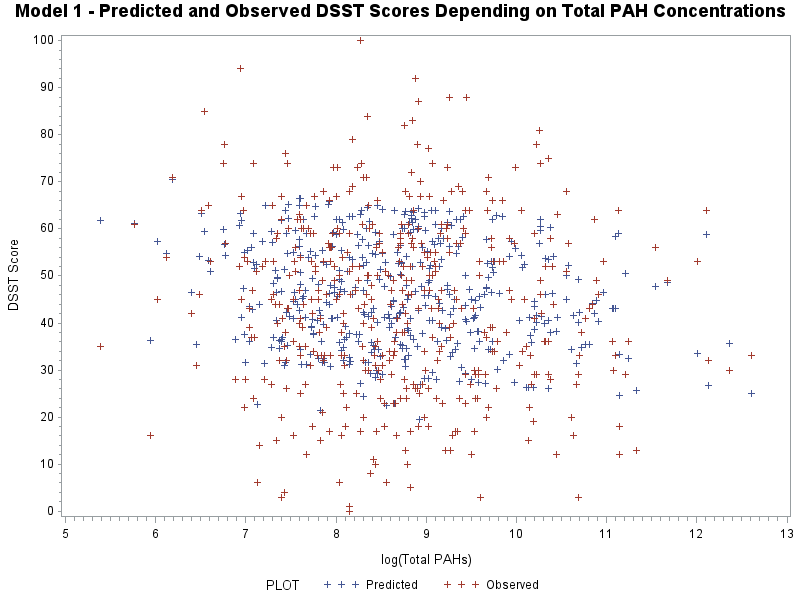


**Plot 4:** Predicted and observed DSST scores compared to age


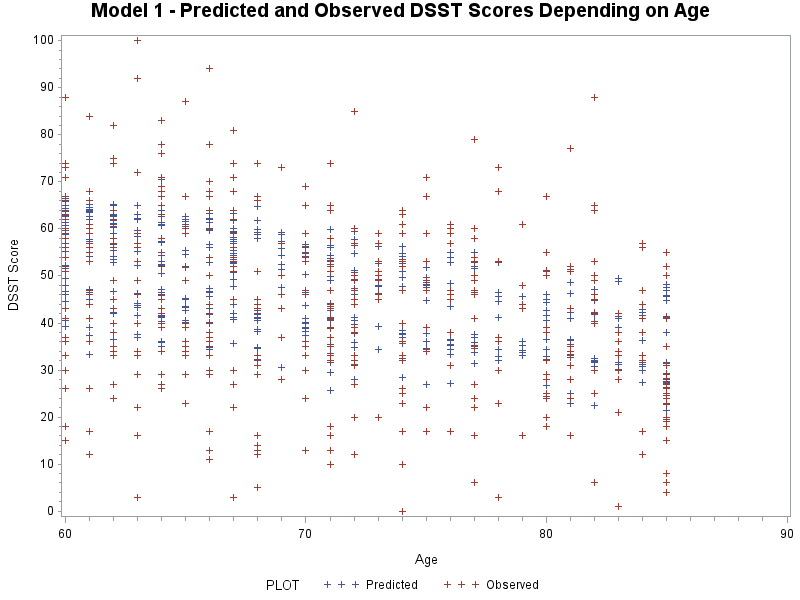


**Model 2: DSST scores compared to summation of naphthol, fluorenol, and phenanthrol (NFP) concentrations, log transformed, and adjusted for age, socio-economic status, and diabetic status**

**Plot 5**: Distribution and probability plot, in normal quantiles, for model residuals


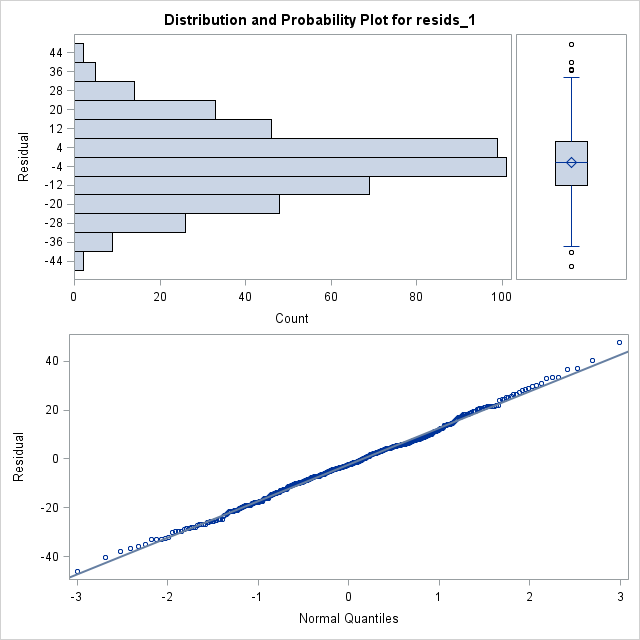


**Plot 6:** Model residuals compared to model predicted values


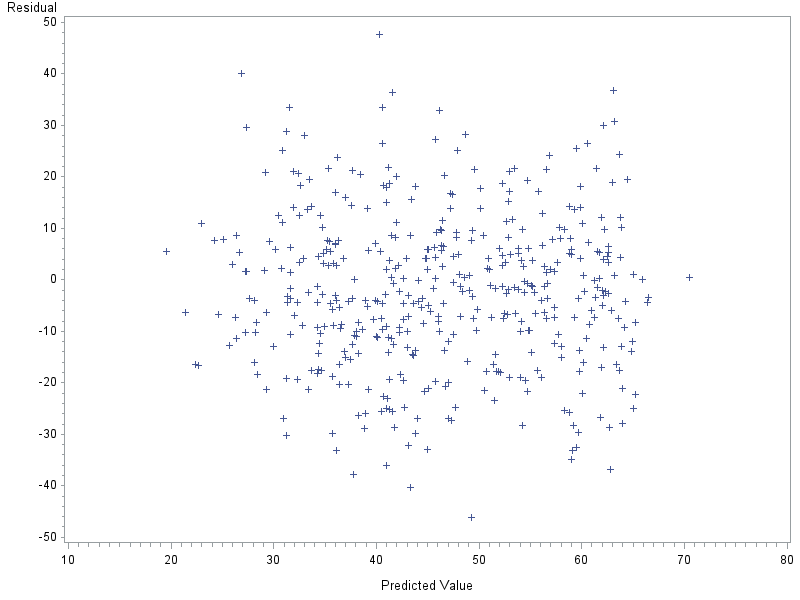


**Plot 7:** Predicted and observed DSST scores compared to log transformed NFP concentrations


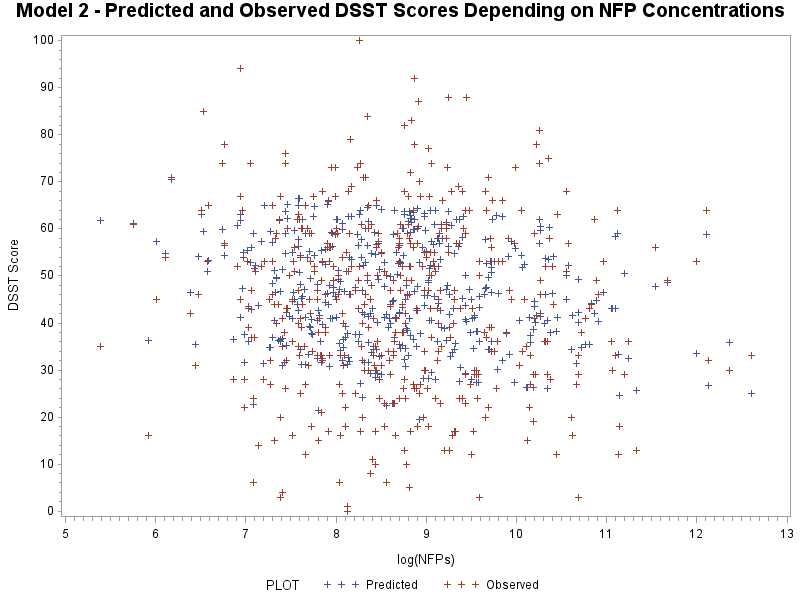


**Plot 8:** Predicted and observed DSST scores compared to age


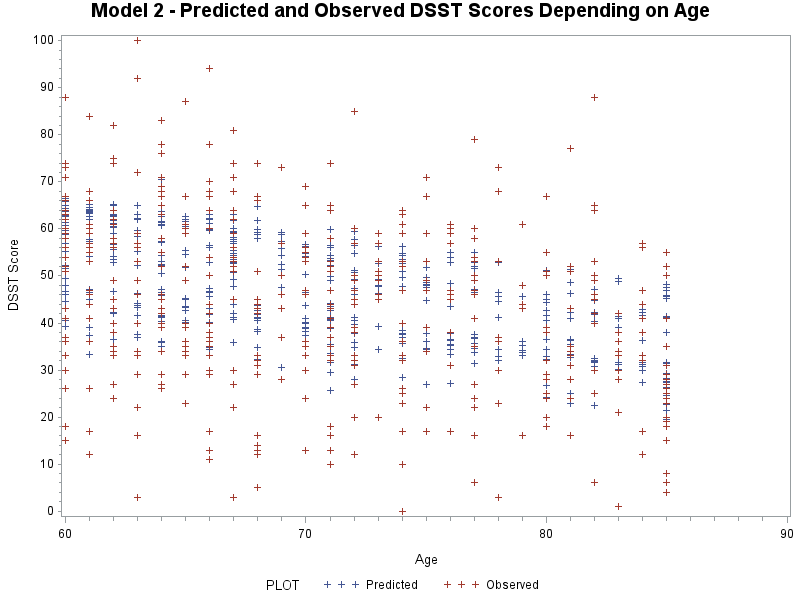


**Model 3: DSST scores compared to 1-hydroxypyrene (1-OHPyr) concentrations, log transformed, and adjusted for age, socio-economic status, and diabetic status**

**Plot 9:** Distribution and probability plot, in normal quantiles, for model residuals


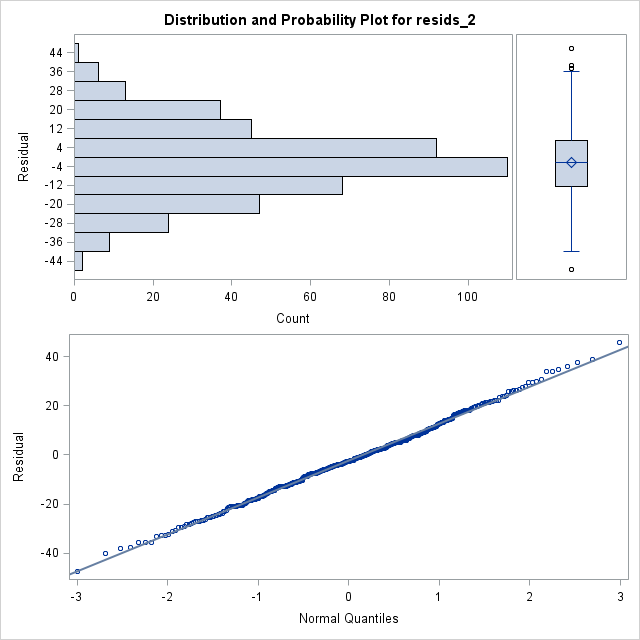


**Plot 10:** Model residuals compared to model predicted values


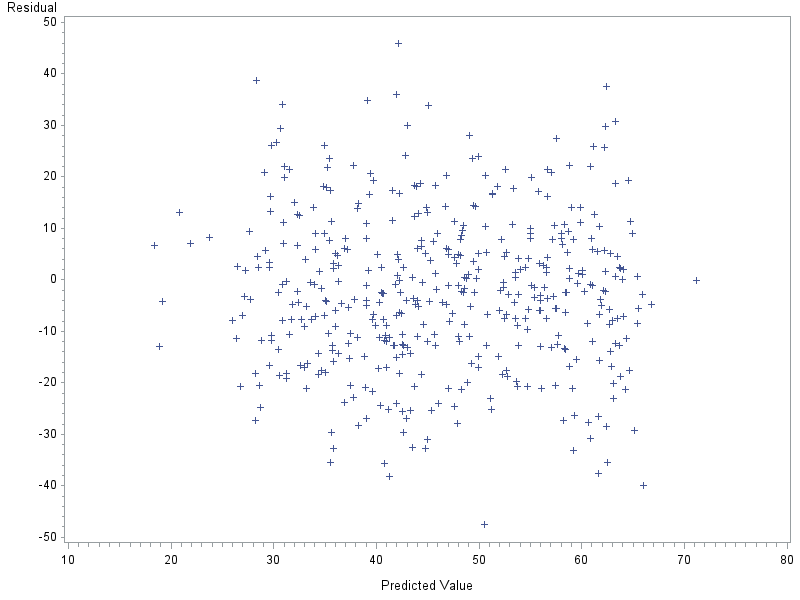


**Plot 11:** Predicted and observed DSST scores compared to log transformed 1-OHPyr concentrations


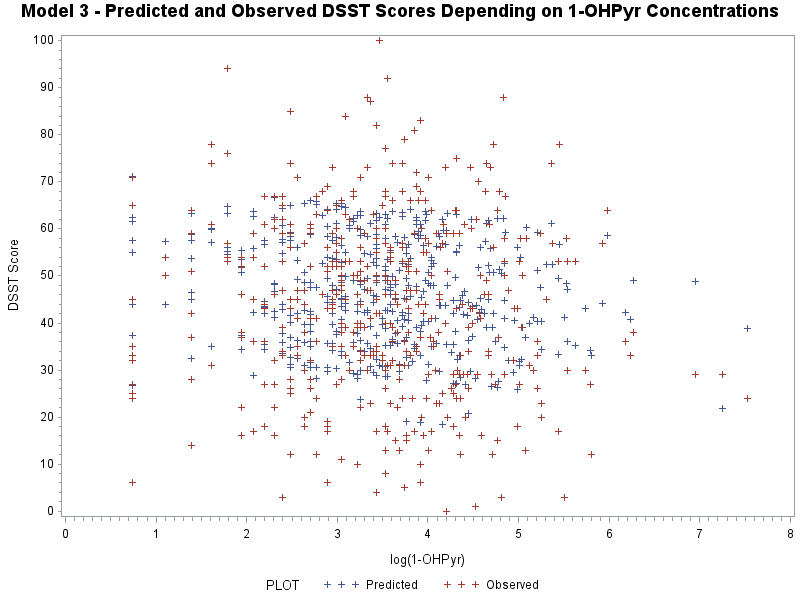


**Plot 12:** Predicted and observed DSST scores compared to age


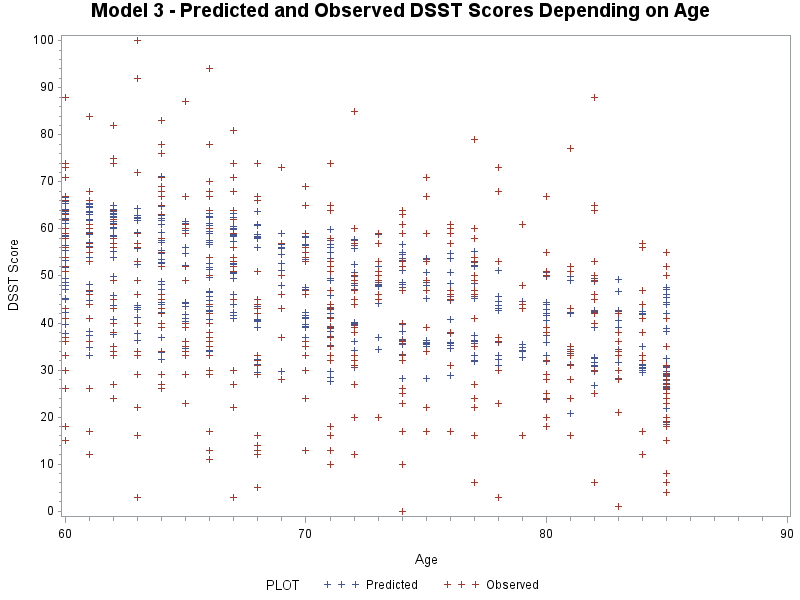

Supplement: S2 Appendix — (DOCX) [file pone.0147632.s002.docx]
